# Porcine reproductive and respiratory syndrome virus triggers mitochondrial fission and mitophagy to attenuate apoptosis

**DOI:** 10.18632/oncotarget.10817

**Published:** 2016-07-24

**Authors:** Shuaifeng Li, Jiaxing Wang, Ao Zhou, Faheem Ahmed Khan, Lin Hu, Shujun Zhang

**Affiliations:** ^1^ Key Laboratory of Agricultural Animal Genetics, Breeding and Reproduction of Ministry of Education, Huazhong Agricultural University, Wuhan, China

**Keywords:** PRRSV, mitochondrial fission, mitophagy, apoptosis, Pathology Section

## Abstract

Porcine reproductive and respiratory syndrome virus (PRRSV) causes acute mitochondrial dysfunction by elevating the level of reactive oxygen species. Mitochondrial dynamics and mitophagy are essential for the maintenance of mitochondrial homeostasis. Here we show that PRRSV infection stimulated mitochondrial fission and mitophagy to attenuate apoptosis in Marc145 cells. PRRSV infection induced the expression of Drp1, enhanced phosphorylation of Drp1 at Ser616 and its subsequent translocation to mitochondria. Furthermore, PRRSV infection increased the expression of PINK1 and Parkin and also stimulated the recruitment of Parkin to mitochondria. In addition, a sensitive dual fluorescence vector expressing mito-mRFP-EGFP targeted mitochondria was employed to observe the complete mitophagy by delivering dysfunctional mitochondria to lysosome for degradation. Interfering the expression of Drp1 and or Parkin suppressed PRRSV replication. More importantly, silencing of Drp1 or Parkin caused significant elevation in apoptotic signaling. These results suggest that PRRSV infection stimulates mitochondrial fission and mitophagy to facilitate virus replication most probably by attenuating apoptosis.

## INTRODUCTION

Porcine reproductive and respiratory syndrome virus (PRRSV) is the etiologic agent of PRRS that causes reproductive failure in sows, severe respiratory distress and high morbidity and mortality in piglets [1]. PRRSV is a single positive-strand RNA virus of the *Arteriviridae* family that replicates its RNA genome on the endoplasmic reticulum derived membrane structure [2, 3]. PRRSV infection induces mitochondrial dysfunction, leading a collapse of the mitochondrial transmembrane potential and raises the content of reactive oxygen species [4].

Mitochondrial homeostasis is maintained by two interlinked processes, mitochondrial dynamics and mitophagy [5]. Mitochondria are highly dynamic organelles that undergo constant fission and fusion. Mitochondrial fusion involves two sets of key GTPase proteins: the outer membrane mitofusins (Mfn1 and Mfn2) and the inner membrane optic atrophy 1 (OPA1) [6, 7]. Mitochondrial fission is mediated by the recruitment of cytosolic dynamic relative protein 1 (Drp1) to mitochondrial surface [8]. Specifically, Drp1 recruitment to mitochondria is regulated by phosphorylation at Ser616[9]. Besides, mitochondrial fission is promoted by several mitochondrial outer membrane proteins, including mitochondrial fission factor (Mff), mitochondrial fission protein 1 (Fis1), mitochondrial dynamic proteins of 49 and 51kDa (MiD49 and MiD51, respectively) [10–12]. These proteins are coordinate to recruit Drp1 to mitochondrial surface.

In human, the mutations of phosphatase and tensin homolog (PTEN)-induced putative kinase 1 (PINK1) and Parkin are linked to autosomal recessive forms of Parkinson's disease [13, 14]. Later researches have uncovered their functional role in selective elimination of damaged mitochondria, termed as mitophagy [15]. PINK1 is maintained at low level through rapid degradation by mitochondrial protease in the N-terminal rule [16]. However, during mitochondrial stress, PINK1 stabilizes on the mitochondrial outer membrane and recruits cytosolic Parkin to mitochondria [17]. Once translocated to mitochondrial surface, Parkin mediates the engulfment and subsequent degradation of mitochondria by autophagosome.

In this study, we investigated the involvement of mitochondrial fission and mitophagy in PRRSV infection. Our results demonstrate that PRRSV infection enhances mitochondrial fission and promotes selective autophagic degradation of damaged mitochondria via mitophagy. PRRSV infection stimulated mitochondrial fission by promoting Drp1 Ser616 phosphorylation and its mitochondrial translocation. PRRSV infection facilitated the elimination of dysfunctional mitochondria via mitophagy. Furthermore, we demonstrated that inhibition of mitochondrial fission and mitophagy by silencing Drp1 and Parkin results in the hindrance of PRRSV replication and enhanced apoptosis. These observations unambiguously implicate the functional relevance of mitochondrial dynamics and mitophagy in PRRSV replication.

## RESULTS

### PRRSV infection promotes mitochondrial swelling and fragmentation

**Figure 1:**
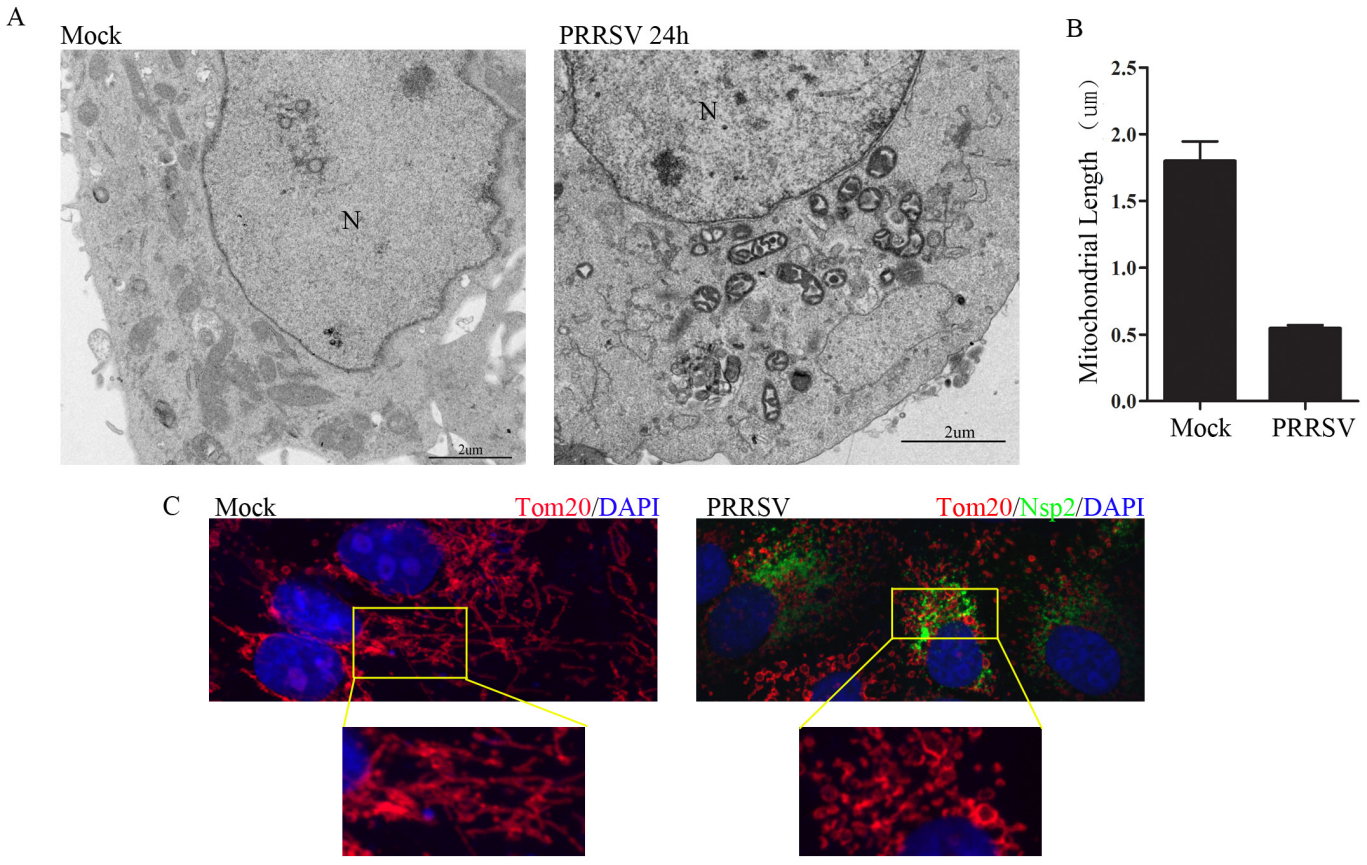
PRRSV infection induces mitochondrial fission **A.** Electron microscopy analysis showing mitochondrial fission in PRRSV-infected cells. At 24h post-infection, PRRSV (MOI, 1) infected Marc145 cells and uninfected cells were examined by electron microscope. In the zoomed images, elongated mitochondria in uninfected cells and fragmented mitochondria with defective cristae in infected cells are shown. **B.** Quantification of mitochondrial diameter is shown. More than 50 cells were calculated for quantification. N, nucleus. **C.** At 24h post-infection, Marc145 cells were immunostained with antibodies specific to tom20 (red), Nsp2 (green). The zoomed images show the typical tubular mitochondria in uninfected cells and fragmented mitochondria in PRRSV infected cells.

PRRSV infection induces oxidative stress of endoplasmic reticulum that causes mitochondrial dysfunction and damage [18, 19]. To investigate the morphological alteration of mitochondria after PRRSV infection, Marc145 cells were infected with PRRSV at a MOI of 1 and were subjected to transmission electron microscopy analysis. As shown in Figure 1A, distinct fragmented mitochondria were observed in PRRSV-infected cells, in contrast to uninfected cells, which displayed elongated mitochondria (Figure 1A). Mitochondrial injury characterized by mitochondrial swelling and cristae distortion was observed after PRRSV infection. A quantitative analysis of relative mitochondrial length after PRRSV infection is presented in Figure 1B. The results from confocal microscopy further substantiated the occurrence of mitochondrial fission in PRRSV-infected cells, compared with uninfected cells (Figure 1C). Together, these results demonstrate that PRRSV infection induces mitochondrial fission.

### PRRSV enhances Drp1 Ser616 phosphorylation and its mitochondrial translocation

**Figure 2:**
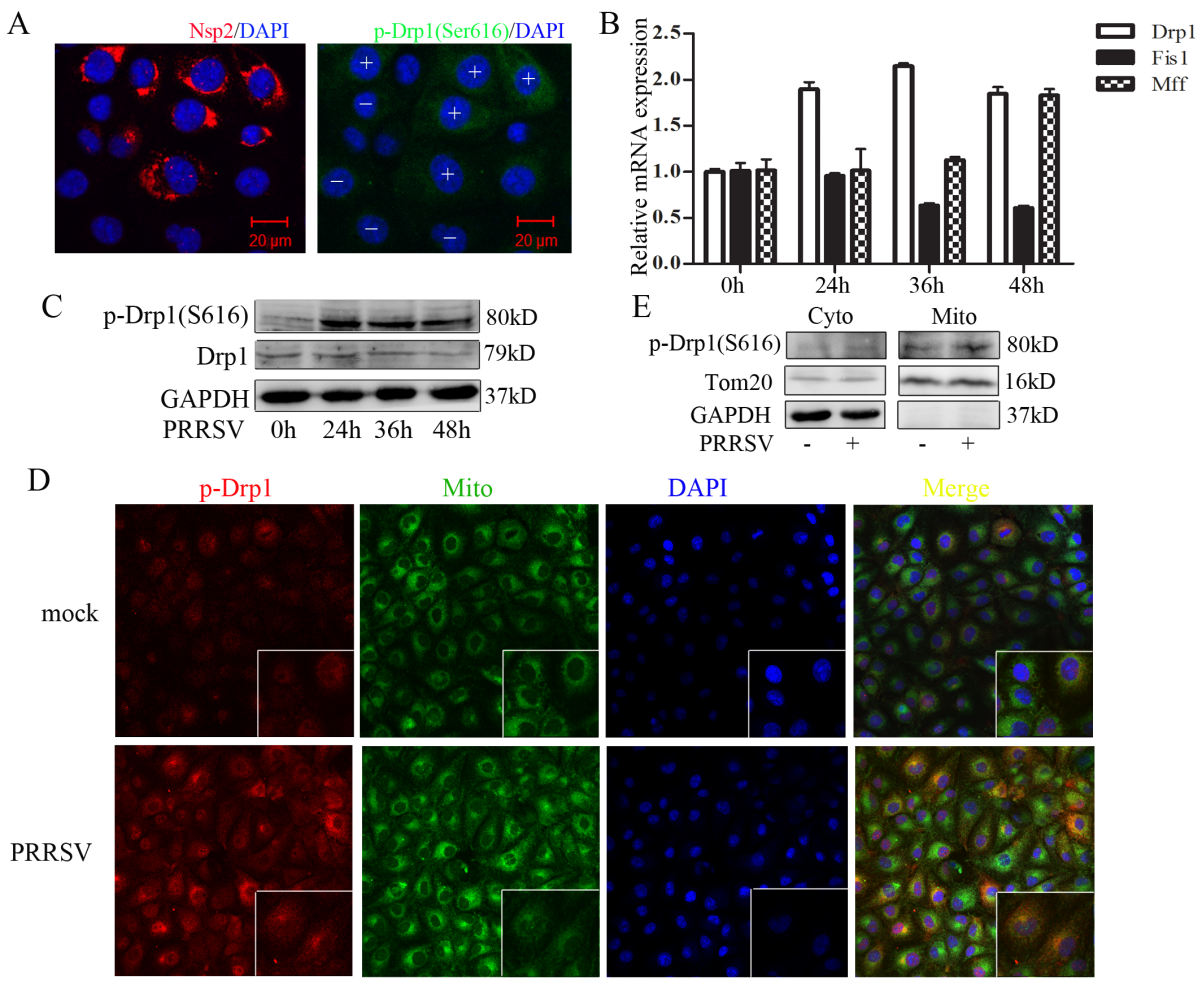
PRRSV infection enhances Drp1 phosphorylation and its mitochondrial translocation **A.** Immunofluorescence analysis showing the induction of drp1 Ser616 phosphorylation in PRRSV-infected cells. +, PRRSV-infected cells; -, uninfected cells. **B.**-**C.** Marc145 cells were infected with PRRSV for 0h, 24h, 36h, 48h, RNA and protein were collected for q-PCR and Weatern Blot analysis. **D.** Marc145 cells were infected with PRRSV for 36h and mitochondria were extracted. Phosphorylated Drp1 of subcellular fraction was determined by Western Blot. Cyto, cytosolic fraction; Mito, mitochondria fraction. Tom20, mitochondria marker; GAPDH, cytoplasmic marker. **E.** Marc145 cells were infected with PRRSV for 36h and location of phosphorylated Drp1was analyzed by confocal microscope. In the zoomed images, the yellow spots indicate the merge of phosphorylated Drp1 with mitochondria. Data are expressed as means with SEM of three independent experiments.

Drp1 recruitment to mitochondria is modulated by phosphorylation at Ser616 by cyclin B/cyclin-dependent kinase 1 (Cdk1) [9, 20]. To investigate whether PRRSV infection enhances Drp1 Ser616 phosphorylation and its mitochondrial location, PRRSV-infected cells were observed by confocal microscopy. We found a significant elevated phosphorylated Drp1 in PRRSV-infected cells as compared to uninfected cells (Figure 2A). Confocal microscopy results also revealed that most of the Ser616 phosphorylated Drp1 translocated to the mitochondria (Figure 2D). PRRSV infection increased the expression of Drp1 and Mff at mRNA level (Figure 2B). However, Fis1 expression was decreased by PRRSV infection (Figure 2B). Although, the total Drp1 protein expression has no change, the Ser616 phosphorylated Drp1 was robustly elevated (Figure 2C). To further substantiate the observation that PRRSV induced Drp1 translocation to mitochondria, we extracted mitochondria from PRRSV-infected and uninfected Marc145 cells. Western Blot analysis of subcellular fractions demonstrated that phosphorylated Drp1 is dramatically enriched in mitochondrial fraction in PRRSV infected cells (Figure 2E). Together, these results indicate that PRRSV promotes mitochondrial fission via Drp1 phosphorylation and its mitochondrial translocation.

### PRRSV induces mitochondrial DNA (mtDNA) depletion and Parkin-dependent mitophagy

**Figure 3:**
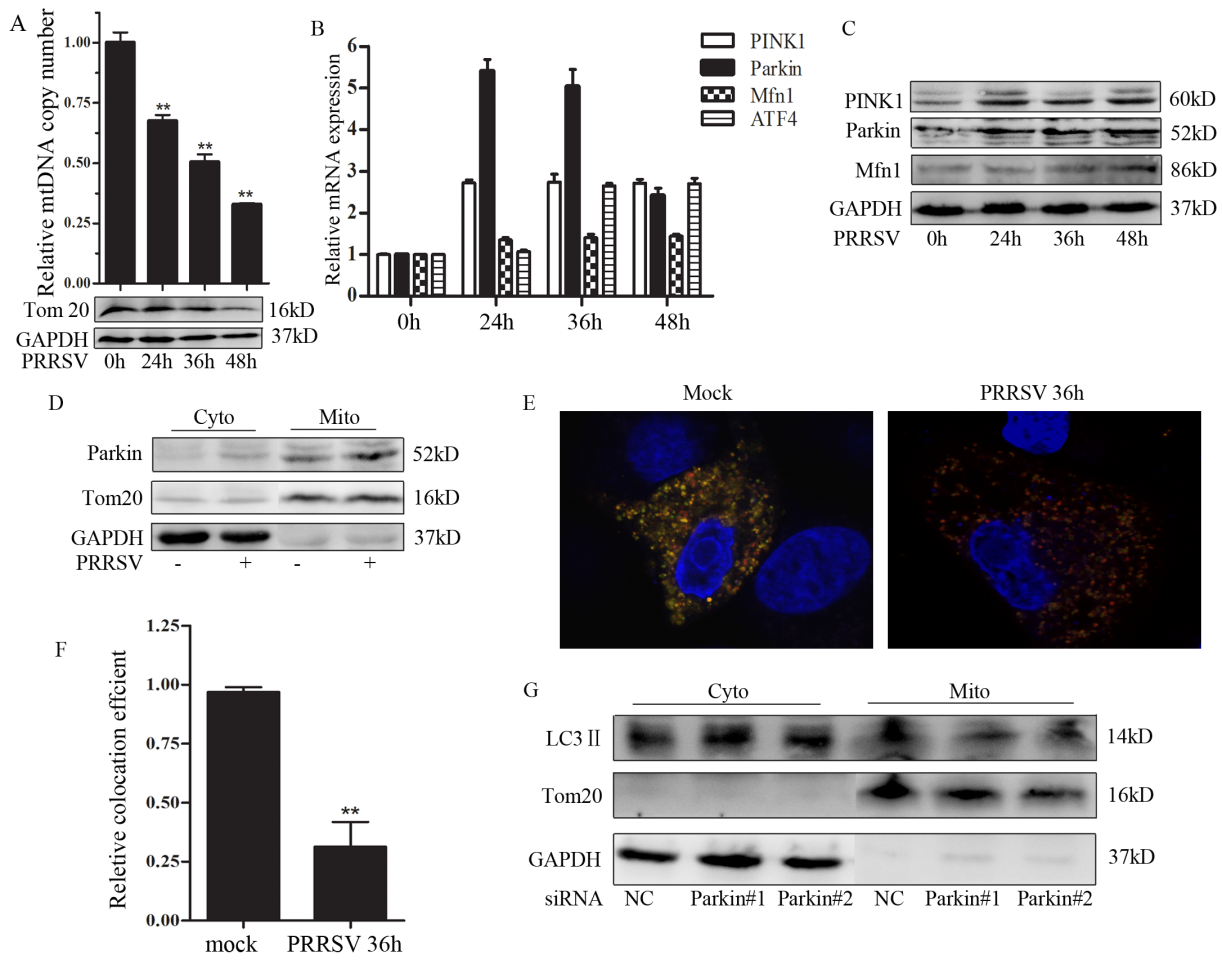
PRRSV induces mtDNA depletion and Parkin-dependent mitophagy **A.** Marc145 cells were infected with PRRSV, DNA and protein were collected at different time point and subjected to q-PCR and Western Blot analysis. **B.** and **C.** Marc145 cells were infected with PRRSV, RNA and protein were collected at different time point and subjected to q-PCR and Western Blot analysis, respectively. **D.** Western Blot analysis the expression of Parkin of the subcellular fraction from uninfected- and PRRSV-infected Marc145 cell. **E.** Marc145 cells were transfected with mito-mRFP-EGFP and infected with PRRSV for 36h. Confocal image analyzed the level of mitophagy. **F.** Quantitative analysis of the fluorescence signal in F. More than 50 cells were calculated for the quantification. **G.** Marc145 cells were transfected with siParkin and infected with PRRSV for 36h. Then mitochondria were separated and the expression of LC3 II was verified by Western Blot. Cyto, cytosolic fraction; Mito, mitochondria fraction. Tom20, mitochondria marker; GAPDH, cytosolic marker. Data are expressed as means with SEM of three independent experiments.

To investigate the possible effect of PRRSV infection on mtDNA copy, we quantified the relative mtDNA copy number as the ratio of mtDNA cytochrome c oxidase subunit I (COI) to 18S rDNA [21]. As shown in Figure 3A, the total number of mitochondria was drastically diminished in PRRSV-infected cells as compared to the uninfected group, which is in line with the result from Tom20 expression. Considering that mitochondria are excluded from cell by mitophagy, we further investigated whether PRRSV stimulates the expression of mitophagy-related genes. PRRSV infection modestly but significantly increased the expression of Parkin and PINK1 at both mRNA and protein levels (Figure 3B and 3C). We have previously shown that PRRSV up-regulated LC3 II expression [22], which is a reliable indicator of autophagic process. Activating transcription factor 4 (ATF4) was also stimulated by PRRSV infection (Figure 3B). ATF4 is a transcriptional factor that triggers Parkin expression via unfolded protein response (UPR) [23]. Parkin translocates to mitochondria is a well-characterized event that triggers mitophagy [24]. To further assess PRRSV-induced mitophagy, we utilized purified mitochondria and cytosolic fraction of PRRSV-infected and uninfected-Marc145 cells to evaluate Parkin translocation. Western Blot analysis of these fractions is presented in Figure 3D. Parkin was markedly enriched in the mitochondrial fraction of PRRSV-infected cells. To monitor the complete mitophagy in PRRSV-infected cells, a dual fluorescence vector encoding a mitochondrial targeting sequence fused with mRFP and EGFP was used. We observed predominantly a yellow color in uninfected cells indicating the mitochondria in cytoplasm, whereas in PRRSV-infected cells, most of the mitochondria displayed red color which is the representative of the delivery of damaged mitochondria to lysosome (Figure 3E). Relative quantification of the co-localization of mRFP and EGFP is shown in Figure 3F. To further substantiate that PRRSV-induced mitophagy is Parkin-dependent, we assessed LC3 II expression in cytosolic and mitochondrial fraction in Parkin silencing cells. The results showed that LC3 II markedly decreased in mitochondrial fraction in Parkin silencing cells (Figure 3G). These results suggest that Parkin-dependent mitophagy was induced by PRRSV infection.

### Drp1 and Parkin deficiency affects mtDNA copy and depresses PRRSV replication

**Figure 4:**
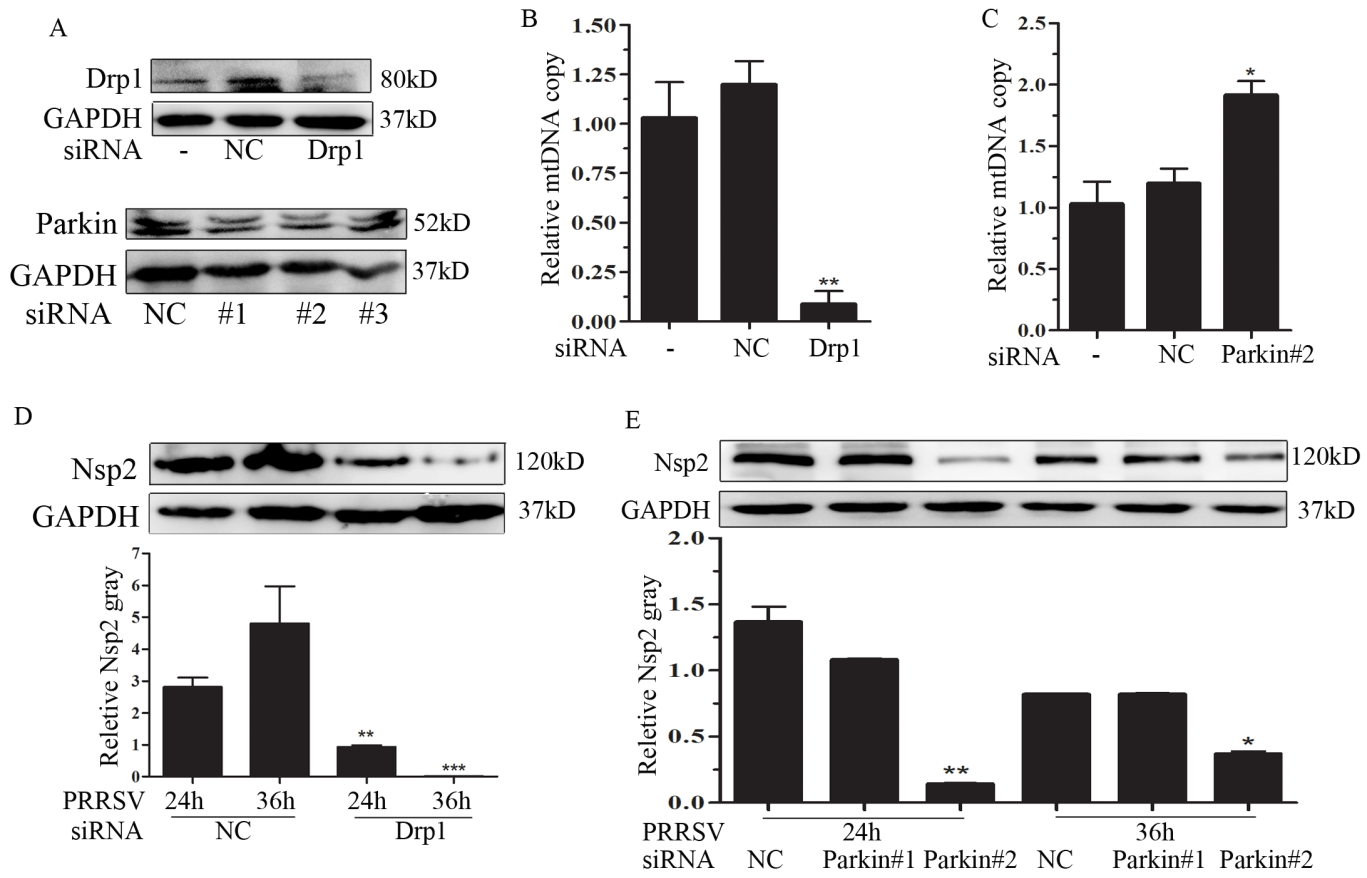
Drp1 and Parkin affects mitochondrial copy and PRRSV replication **A.** Marc145 cells were transfected with negative control (NC) or specific siRNA targeting Drp1 or Parkin. At 24h post-transfection, the silencing effect was determined. **B.** and **C.** The DNA were extracted from Mock-, NC-, Drp1- and Parkin-transfected cells and subjected to mitochondrial copy determination by qPCR. **D.** and **E.** The effect of Drp1 and Parkin silencing on PRRSV replication were determined by Western Blot. Nsp2 protein gray was quantified by Image J. Data are expressed as means with SEM of three independent experiments. *, *P* < 0.05; **, *P* < 0.01; ***, *P* < 0.001.

To evaluate the functional role of Drp1-dependent mitochondrial fission and Parkin-dependent mitophagy in mtDNA copy and PRRSV replication, we employed the siRNA strategy to silence the expression of Drp1 and Parkin. We first verified the silencing efficiency of Drp1 and Parkin by Western Blot (Fig 4A). Among the three siRNAs of Parkin, siRNA #1 and siRNA #2 were effective, so we chose them for further investigation. It has been shown that PRRSV infection resulted in the depletion of mtDNA copies (Figure 3A). Here, we further examined the effect of silencing Drp1 or Parkin on PRRSV-induced mtDNA depletion. Interference of Drp1 expression profoundly exacerbated PRRSV-induced mtDNA depletion (Figure 4B). Whereas, silencing of Parkin robustly blocked PRRSV-induced mtDNA depletion (Figure 4C). To characterize the functional significance of Drp1 and Parkin in PRRSV replication, we determined PRRSV replication in Drp1- or Parkin-silencing cells. As shown in Figure 4D and 4E, intracellular viral non-structural protein 2 (Nsp2) was decreased in Drp1- or Parkin-deficient cells, indicating the functional role of Drp1-dependent mitochondrial fission and Parkin-dependent mitophagy in PRRSV replication.

### Disruption of mitochondrial fission and mitophagy induces apoptosis

**Figure 5:**
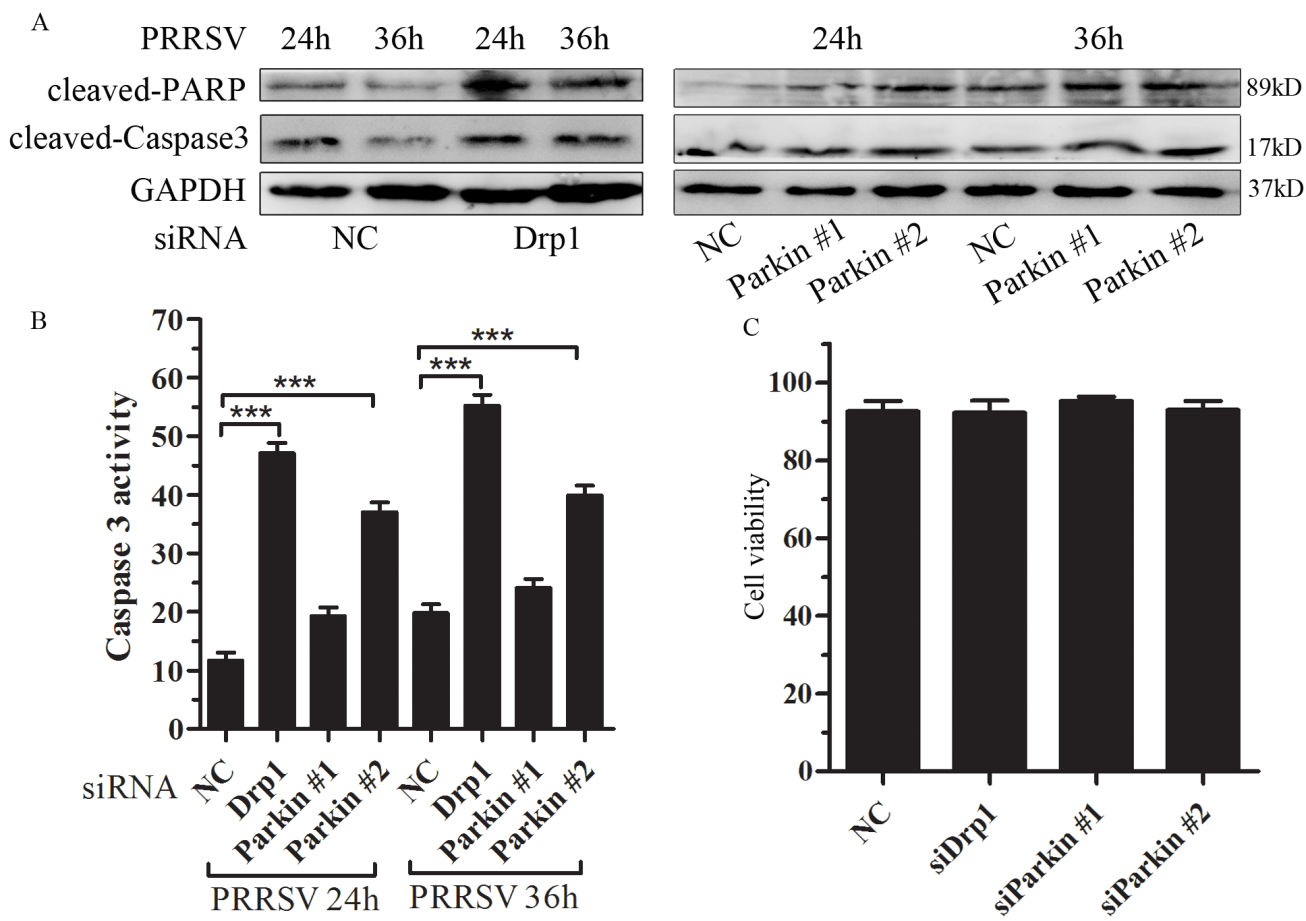
Disruption of mitochondrial fission and mitophagy lead to induction of apoptosis **A.** and **B.** Drp1 and Parkin silencing accelerates PRRSV induced apoptosis. Marc145 cell were transfected with negative control (NC) or specific targeting Drp1 or Parkin and infected with PRRSV. At 24h and 36h postinfection, apoptosis was analyzed by Western Blot (A) and Caspase 3 activity (B). **C.** Marc145 cells were seeded in 12-well plate for 24h and transfected with indicated siRNAs for other 24h, cells were collected and detected cell viability by trypan blue straining. Data are expressed as means with SEM of three independent experiments. ***, *P* < 0.001.

Mitochondrial dynamic is closely linked to apoptosis [25, 26]. Caspases are a family of cysteine proteases that play a vital role in apoptosis and inflammation, demonstrated by their cleavage activation [27]. Depletion of Drp1 or Parkin induced robust activation of caspase 3 and substantial cleavage of poly (ADP-ribose) polymerase (PARP), a substrate of caspase 3 (Figure 5A). Induction of apoptosis was also substantiated by caspase 3 activity assay (Figure 5B). In order to exclude the impact of siRNAs on cell viability, cell viability was tested by trypan blue exclusion. The results indicated that there was no significant difference on cell viability between NC-transfected cells and Drp1- or Parkin-transfected cells (Figure 5C). Together, these results strongly suggest that PRRSV-mediated induction of mitochondrial fission and mitophagy, although serving as a quality control mechanism to eliminate dysfunctional mitochondria, also protects PRRSV-infected cells from apoptotic cell death, and facilitating persistent viral infection.

## DISCUSSION

The mitochondrial dynamics and mitophagy play crucial roles in metabolism, cellular differentiation and neurodegeneration [28, 29]. In the past, the aspects of mitochondrial dynamics and mitophagy were broadly studied in the context of neurodegenerative disorders like Parkinson's and Huntington's diseases [28]. The study of the essential role of mitochondrial dynamic and mitophagy in virus infection is in its infancy. Our study characterized for the first time that PRRSV usurps Drp1-mediated mitochondrial fission to promote viral replication. A similar result was also observed in Hepatitis C virus [30] and Hepatitis B virus [31]. It may provide a potential mechanism by which PRRSV exploit mitochondrial fission in favor to viral replication. However, the molecular mechanism by which PRRSV induces mitochondrial fission needs further investigation.

Many DNA and RNA viruses have been shown to tightly regulate the autophagy process to prevent their clearance by host cell, to evade host immune response, or to favor its replication [32]. Recently, several researches including our group have been shown that PRRSV facilitates bulk autophagy to favor its replication [22, 33–35]. Several viruses including Hepatitis C virus [36], Oncolytic measles virus [37] and Newcastle disease virus [38] have been confirmed to subvert mitophagy to facilitate their own proliferation. Here, we found that PRRSV infection induced Parkin-dependent mitophagy (Figure 3). siRNA-mediate interference of Parkin mitigated PRRSV proliferation (Figure 4E). Our data suggests a functional interaction between PRRSV and autophagy pathway providing evidence that selective autophagy is probably involved in promoting PRRSV replication. Previous study has demonstrated that NOD-RIP2 signaling was inducible during PRRSV infection [39]. The research from Lupfer et al. have indicated that NOD-RIP2 signaling play an essential role in regulation of Influenza A virus-induced mitophagy, which exclude the role of Parkin in this process [40]. This suggests that diverse mitophagy signaling is exploited by viruses to their own benefits. Illumination of the functional relevance of mitophagy and virus replication will provide new therapeutic strategies to resist virus infection.

Apoptosis is considered to be an essential mechanism of host defense to limit viral replication [41]. The results of the study reported herein support the idea that mitochondrial dynamics and mitophagy attenuate apoptosis during virus infection. Our results demonstrated that PRRSV-induced apoptosis was drastically elevated when the cell suffered from mitochondrial fission or mitophagy depletion (Figure 5). One possible mechanism is that the segregation and degradation of damaged mitochondria resulting from PRRSV infection by mitochondrial fission and mitophagy may delay the induction of apoptosis by preventing the release of cytochrome C. In summary, this study provides evidence that PRRSV hijacks host signaling pathway by inducing mitochondrial fission and mitophagy to facilitate persistent viral infection which extend our knowledge about the pathology of PRRSV infection.

## MATERIALS AND METHODS

### Cells and virus

Marc145 cells that is a clone of the African green monkey kidney cell line MA-104, were obtained from China Center for type Culture Collection (CCTCC) and cultured in Dulbecco's modified Eagle's medium (DMEM) supplemented with 10% fetal bovine serum (FBS), 100 U/ml penicillin, and 100 g/ml streptomycin at 37 °C in a humidified 5% CO_2_ incubator. PRRSV strain WUH3, which was adapted to Marc145 cells at a MOI of 0.5. Virus stocks of PRRSV were prepared in Marc145 cells and infectious virus titer was analyzed in Marc145 cells using the Reed and Muench method [42]. For virus infection, cells were initially incubated with PRRSV for 1 h at 37 °C. After 1 h of adsorption, cells were exchanged to the medium containing 5% FBS and cultured for indicated time.

### DNA constructs

To construct mito-mRFP-EGFP plasmid, the sequence of human cytochrome c oxidase subunit VIII (COXVIII) was inserted N terminally in frame into pMCS-mRFP-EGFP (Yrgene). The primer used for amplification is as follows: COXVIII former primer 5′- GAAGATCTATCATGTCCGTCCTGACGCCGCTGC-3′, COXVIII reverse primer 5′- CGGAATTCACACTCTGGCCTCCTGTAGGTCTCC-3′.

### Quantitative real-time PCR

Total cellular RNA were extracted from cells using Total RNAeasy kit (Omega, R6834-01), subsequently, complementary DNAs (cDNAs) were synthesized by using First-Strand Synthesis System (Thermo Scientific, #00238582) according to the manufacturer's instructions. To analyze the expression level of mitochondrial DNA, total cellular DNA was extracted from the cells using DNA kit (Qiagen, M1116) and subsequently quantified by qPCR. All PCR was performed under the following conditions: 95°C for 3 min followed by 40 cycles of 95°C for 10s, 60°C for 20s. SYBR Premix ExTaqTM kit (Bio-Rad, #172-5121) was used according to the manufacturer's instructions, and the real-time PCR was performed on an iQ5 Real-Time PCR system (Bio-rad). The real-time PCR results were analyzed and expressed as relative expression of CT (threshold cycle) valuing the 2^−^ΔΔ^Ct^ method. All primer pairs used for RT-PCR are presented in Table 1.

**Table 1:** Primers used for quantitative real-time PCR

gene name	primer name	primer sequence (5′-3′)
Drp1	F1	TGGGCGCCGACATCA
	R1	GCTCTGCGTTCCCACTACGA
Fis1	F1	GTCTGTGGAGGACCTGCTGA
	R1	CACGATGCCTTTACGGATG
Mff	F1	GCACTGAAAACACCACCTCG
	R1	TCTGCCAACTGCTCGGATT
Parkin	F1	GCCACCTACCCAGTGACCAT
	R1	TCGCTTAGCAACCACCTCCT
PINK1	F1	GGCTTGTCAATCCCTTCTACG
	R1	AGCACATTTGCGGCTACTC
ATF4	F1	CGATTCCAGCAAAGCACC
	R1	ATCCACAGCCAGCCATTC
COI	F1	CTAACAGACCGCAACCTCAAC
	R1	TCCGAAGCCTGGTAGGATAAG
18S rRNA	F1	TGTGATGCCCTTAGATGTCC
	R1	TGGGGTTCAGCGGGTTAC
Beta actin	F1	AGCAAGCAGGAGTATGACGAGT
	R1	CAAGAAAGGGTGTAACGCAACT

### Western blot

The whole cell lysate were extracted with RIPA buffer containing 1% protease inhibitor PMSF, 1% cocktail (SIGMA, P8340) and 1% phosphatase inhibitor (SIGMA, P0040). Mitochondria were separated by using a Mitochondria/Cytosol Fractionation Kit (Beyotime, C3601), according to the instruction. 10-12% SDS-PAGE gels was used to separate proteins and then transferred to polyvinylidene fluoride (PVDF) membranes (Bio-rad). The membranes were blocked with 5% nonfat milk and incubated with the primary antibody overnight at 4°C. Membranes were washed and incubated with HRP-conjugated secondary antibody. The proteins were detected by the enhanced chemiluminescence (ECL) system (Bio-Rad) following the manufacturer's instructions. The relative expression normalized GAPDH was calculated in Image J software (NIH).

### Confocal fluorescence microscopy

The Marc145 cells were grown on glass cover slips and transfected with indicated plasmid DNA, fixed in 4% paraformaldehyde, washed, and then permeabilized with 0.1% TritonX-100. The cells were incubated with the indicated antibodies. Wherever indicated, nuclei are stained with DAPI. Images were visualized under a 63× or 100× oil objective using a confocal microscope.

### Electron microscopy

Briefly, Marc145 cells and PRRSV-infected cells grown in 35mm dishes were washed and fixed with fixative containing 2% glutaraldehyde in 0.1M sodiun cacodylate buffer. Cell pellets were embedded in 2% agarose, post-fixed with 1% osmium tetroxide, and dehydrated with an ethanol series. Samples were infiltrated, embedded in Durcupan and polymerized at 60°C for 48h. Ultrathin sections were prepared and examined using Tecnai transmission electron microscope.

### Cell viability assay

Cell viability was determined by trypan blue exclusion assay. Marc145 cells were transfected with indicated siRNA. At 24h post-transfection, cells were harvested by Trypsin/EDTA and stained 0.4% trypan blue staining solution.

### Reagents and antibodies

Chemical reagents used in this study were Mito Green Tracker (Beyotime, C1048). Primary antibodies used in this study include the following: rabbit polyclonal anti-Drp1 (Proteintech, #12957-1-AP, 1: 500 dilution); rabbit polyclonal anti-Drp1(Ser616) (Cell Signaling, #3455, 1:1000 dilution); rabbit polyclonal anti-PINK1 (Cell Signaling, #6946, 1:1000 dilution); rabbit monoclonal anti-Parkin (abcam, ab15954, 1:1000 dilution); rabbit polyclonal anti-Mfn1 (Proteintech, #13798-1-AP, 1: 500 dilution); rabbit monoclonal anti-LC3 II (Cell Signaling, #3868, 1:1000 dilution); rabbit anti-cleaved Caspase3 (Cell Signaling, #9664, 1:500 dilution); rabbit anti-cleaved PARP (Cell Signaling, #9509, 1:500 dilution); mouse monoclonal anti-GAPDH (Proteintech, #60004-1-lg, 1:1000 dilution); rabbit polyclonal anti-Tom20 (Proteintech, #11802-1-AP, 1:1000 dilution); mouse polyclonal anti-PRRSV Nsp2 has been described previously [39]. The secondary antibodies used for immunoblot analysis were HRP-conjugated anti-mouse IgG (BOSTER), HRP-conjugated anti-rabbit IgG (Cell Signaling). The secondary antibodies used for immunofluorescence was Cy3-conjugated anti-mouse IgG (BOSTER), FITC-conjugated anti-mouse IgG (BOSTER).

### Transfection of siRNA

SiRNA was transfected into Marc145 cells at the concentration of 100pM with Lipofectamine 2000 (Invitrogen, 11668-019) according to the manufacturer's protocol. SiRNA is synthesized by Gene-Pharma (Shanghai, China). The siRNA sequences are as follows: siDrp1: 5′-AACGCAGAGCAGCGGAAAGAG-3′ [43]; siParkin #1: 5′-GGUAGAUCAAUCUACAACATT-3′; siParkin #2: 5′-CCUGAUCGCAACAAAUAGUTT-3′; siParkin #3: 5′-CCUUCUGCCGGGAAUGUAATT-3′; Negative control (NC): 5′-UUCUCCGAACGUGUCACGUTT-3′.

### Statistical analysis

All experiments were performed independently three times, and data were presented as the means with SEM. Data were analyzed by One-way ANOVA followed by Tukey's Honest Significant difference test with SPSS version 16.0 (SPSS, Chicago, IL, USA). *P* value < 0.05 was considered as statistically significant.
